# Whole-exome sequencing identifies a *de novo TUBA1A* mutation in a patient with sporadic malformations of cortical development: a case report

**DOI:** 10.1186/1756-0500-7-465

**Published:** 2014-07-22

**Authors:** Keiko Shimojima, Aya Narita, Yoshihiro Maegaki, Akira Saito, Toru Furukawa, Toshiyuki Yamamoto

**Affiliations:** 1 Precursory Research for Embryonic Science and Technology (PRESTO), Japan Science and Technology Agency (JST), Kawaguchi 332-0012, Japan; 2 Tokyo Women’s Medical University Institute for Integrated Medical Sciences, Tokyo 162-8666, Japan; 3 StaGen Co., Ltd., Tokyo 111-0051, Japan; 4 Division of Child Neurology, Faculty of Medicine, Tottori University, Yonago 683-8504, Japan

**Keywords:** Whole-exome sequencing, *TUBA1A*, Malformations of cortical development, Lissencephaly, Microcephaly

## Abstract

**Background:**

Owing to the number of genetic mutations that contribute to malformations of cortical development, identification of causative mutations in candidate genes is challenging. To overcome these challenges, we performed whole-exome sequencing in this study.

**Case presentation:**

A Japanese patient presented with microcephaly and severe developmental delay. Brain magnetic resonance imaging showed the presence of colpocephaly associated with lateral ventricle dilatation and the presence of a simplified gyral pattern. Hypoplasia of the corpus callosum and cerebellar vermis were also noted. Because Sanger sequencing is expensive, laborious, and time-consuming, whole-exome sequencing was performed and a *de novo* missense mutation in *TUBA1A* (E27Q) was identified.

**Conclusion:**

The novel mutation identified in this study was located in the genetic region that encodes the N-terminal domain of TUBA1A, a region of *TUBA1A* with few reported mutations. Retrospective assessment of the clinical and radiological features of this patient―i.e., microcephaly, lissencephaly (pachygyria) with cerebellar hypoplasia, and corpus callosum hypoplasia―indicated that the *TUBA1A* mutation did not lead to any contradictions. Because rapid and comprehensive mutation analysis by whole-exome sequencing is time- and cost-effective, it might be useful for genetic counseling of patients with sporadic malformations of cortical development.

## Background

Malformations of cortical development (MCD) are a cause of motor and intellectual disabilities and severe epilepsy [1]. Although various types of MCD have been described, the most typical type is lissencephaly, a group of malformations caused by abnormal neuronal migration [2]. The gene encoding platelet-activating factor acetylhydrolase isoform 1b regulatory subunit 1 (*PAFAH1B1*, formerly *LIS1*) was initially identified as the causative gene of lissencephaly. Advances in neuroradiological imaging and genetic diagnosis have since improved our understanding of the underlying pathogenesis of such diseases. Mutations in tubulin genes, encoding tubulin alpha 1a (*TUBA1A*), beta 2B class IIb (*TUBB2B*), and beta 3 class III (*TUBB3*), are now recognized as major causes of MCD [3,4]. Moreover, the rapid development of new revolutionary molecular technologies has enabled us to identify rare genetic variants in sporadic MCD cases [5].

In this study, we examined a Japanese patient with sporadic MCD. As mentioned above, causative genes are difficult to identify, owing to the large number of MCD candidate genes. Sanger sequencing of potential causative genes is expensive, laborious, and time-consuming; thus, we performed whole-exome sequencing. Using this method, we identified a novel *TUBA1A* mutation. Here, we discuss the advantages of using whole-exome sequencing for the rapid detection of causative mutations and genetic counseling of patients with sporadic MCD.

## Case presentation

### Patient description

**Figure 1:**
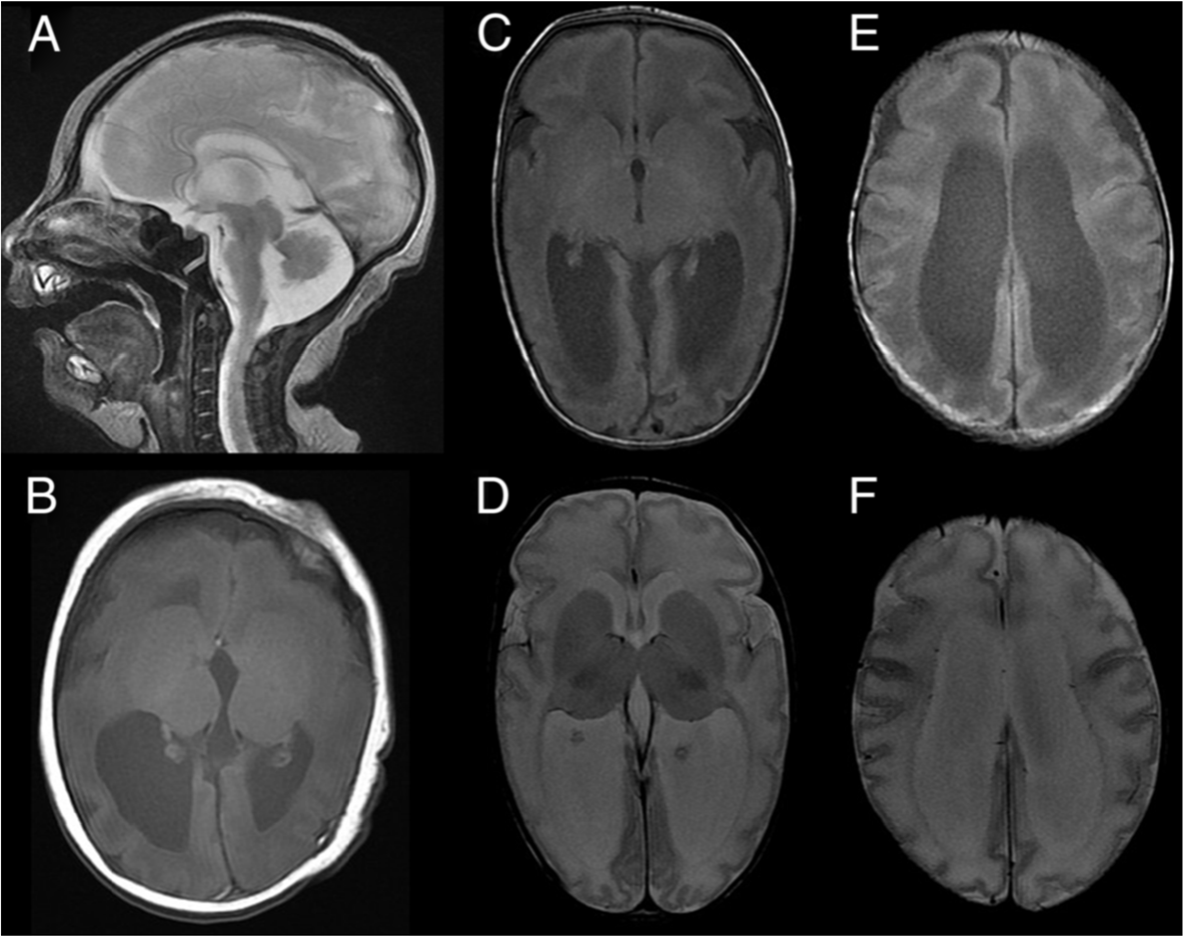
**Brain magnetic resonance imaging of the patient.** T2-weighted sagittal **(A)** and T1-weighted axial **(B)** images at 6 days after birth indicate hypoplastic vermis of the cerebellum, cortical dysgenesis with a simplified gyral pattern, and dilatation of the lateral ventricles. T1-weighted **(C and E)** and T2-weighted **(D and F)** axial images at 3 months of age indicate colpocephalic appearance associated with dilatation of the lateral ventricles. Cortical gyration is poor and reduced volume of the white matter is apparent.

A Japanese baby girl was born at 41 weeks of gestation with a birth weight of 3,400 g (+1.0 standard deviation [SD]), length of 49.5 cm (+0.5 SD), and an occipitofrontal circumference (OFC) of 31.5 cm (-1.0 SD). Her Apgar score was 7/8 (1 min/5 min). Her 30-year-old mother was gravida 0, para 0. At 22 weeks of gestation, fetal microcephaly was detected by echosonography. Soon after delivery, the sucking ability of the infant was good despite mild muscle hypotonia. She showed marked posterior sloping of the forehead, a high-arched palate, and microretrognathia. At 6 days after birth, brain magnetic resonance imaging (MRI) revealed colpocephalic configulation of the lateral ventricle dilatation associated with a simplified gyral pattern (Figure 1).The sucking ability of the infant gradually decreased; at 2 months of age, tube feeding was initiated. At this age, she weighed 4.7 kg (-0.8 SD) and measured 59.4 cm (+1.2 SD) in length, with an OFC of 34.0 cm (-3.3 SD), indicating severe microcephaly. She could not pursue objects. Although her muscle tone was within normal limits, she often showed an opisthotonic posture. At the age of 3 months, brain MRI examination was performed again, and brain hypoplasia associated with diffuse pachygyria and lateral ventricle dilatation was found, which is atypical for lissencephaly patients (Figure 1).

Conventional karyotyping revealed a normal female karyotype with 46,XX. Because this child was the first born, genetic counseling was provided and both parents expressed a desire for their daughter to be genotyped.

We searched for the causative mutation(s) by whole-exome sequencing by using a next generation sequencer. After obtaining written informed consent, we obtained blood samples from the patient and her biological parents. This study was approved by the ethical committee at Tokyo Women’s Medical University.

### Whole-exome sequencing

Genomic DNA collected from the patient and her parents were used as trio samples for whole-exome sequencing. DNA was extracted using a QIAamp DNA extraction kit (Qiagen, Hilden, Germany). Using a SureSelect whole exon kit (Agilent Technologies, Santa Clara, CA), 3 μg of DNA was processed according to the manufacturer’s instructions. Captured DNA molecules were analyzed using a SOLiD3® system by paired-end analysis (Life Technologies, Foster City, CA).

Color space reads were mapped to the UCSC hg19 reference genome using SOLiD® BioScope™ software (version 1.3; Life Technologies). Single-nucleotide variants (SNVs) were subsequently called by the DiBayes algorithm using medium call stringency. Small insertions and deletions (indels) were detected using the SOLiD Small InDel Tool (Life Technologies). Called SNVs and indels were filtered based on depth and quality, combined, and the annotated using ANNOVAR and a custom analysis pipeline [6]. Data for annotations was downloaded from the UCSC database (http://genome.ucsc.edu/).

**Figure 2:**
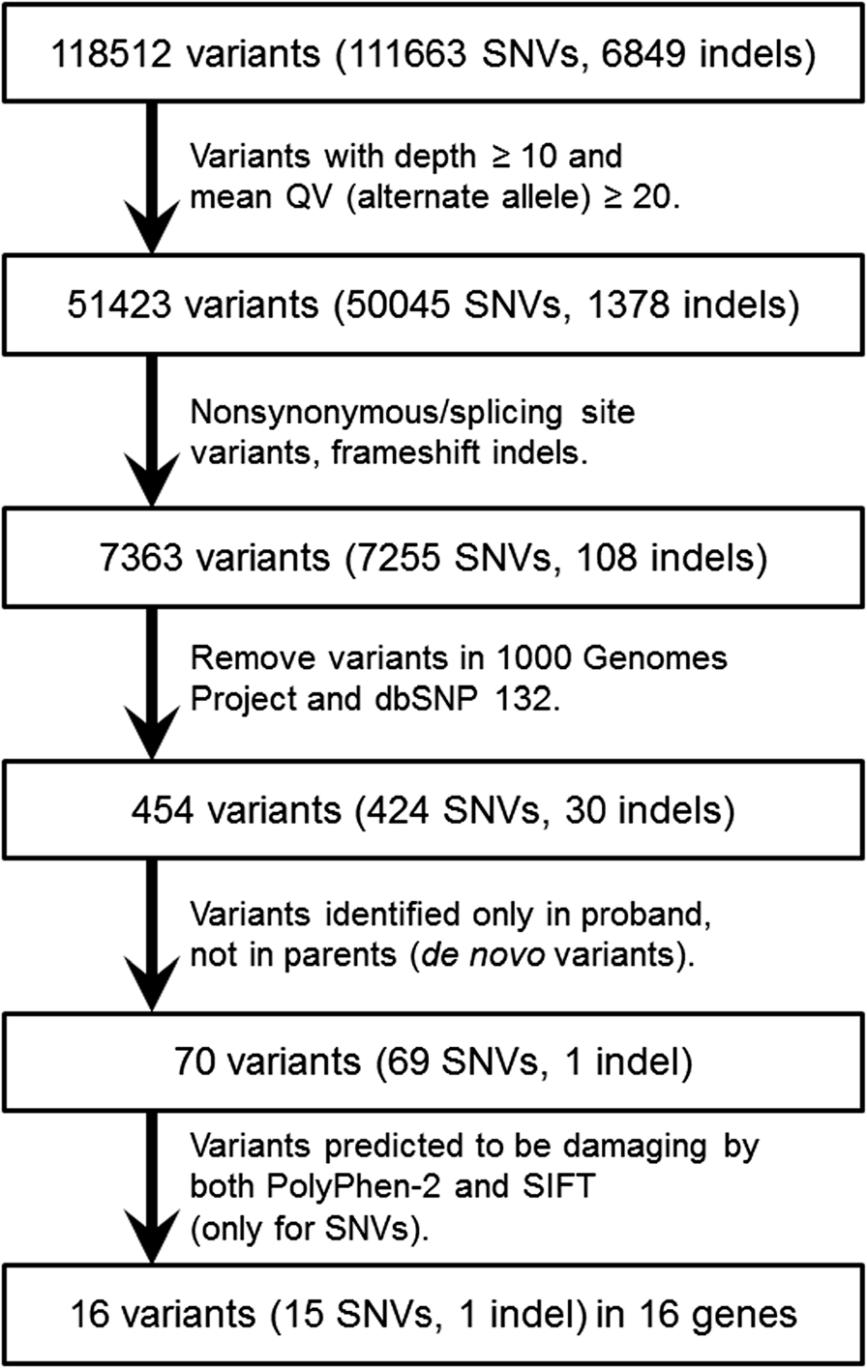
**Flow chart indicating the validation process for variants.** After 5 steps of validation, 16 genetic variants are finally selected.

As a priority, we focused only on non-synonymous coding variants, splice acceptor and donor site mutations, and frameshift indels. We extracted the candidate variants that were not observed in the 1000 Genomes Project (http://www.1000genomes.org/) and the dbSNP 132 database (http://www.ncbi.nlm.nih.gov/projects/SNP/). Because we assumed that an autosomal dominant trait existed in this family, variants identified only in the proband, and not in the unaffected parents (*de novo* variants), were selected. Missense mutations were tested for mutational effects by using amino acid substitution prediction tools such as PolyPhen-2 (http://genetics.bwh.harvard.edu/pph2/) and SIFT (http://sift.jcvi.org/) [7,8]. The flow chart used to select candidate genes is shown in Figure 2.

The potential genetic variants identified by whole-exome sequencing were reconfirmed using standard polymerase chain reaction (PCR)-direct sequencing analysis (Sanger sequencing), as described previously [9,10]. The effects of the identified amino acid changes were analyzed using web-based software such as Pôle Bioinformatique Lyonnais Network Protein Sequence Analaysis (http://doua.prabi.fr/) and PSIPRED Protein Sequence Analysis Workbench (http://bioinf.cs.ucl.ac.uk/psipred/).

Whole-exome sequencing generated an average of 223 × 10^6^ mapped reads that could be mapped to the reference genome—98% could be mapped accurately—with a mean coverage of 93 (Table 1). After filtering, 1 indel and 15 SNVs remained for the *de novo* mutation model (Table 2). For this model, the status of both parents was required to define the wild-type state. No genetic variation remained for the X-linked recessive and autosomal recessive models.

**Table 1:** Whole-exome sequencing output

**Sample**	**Proband**	**Father**	**Mother**
Total reads	406,845,894	437,902,198	396,963,736
Mapped reads	252,829,848	214,184,732	201,932,294
Properly mapped reads	248,030,760	209,714,312	197,748,762
Total bases mapped	9,844,652,145	9,331,887,457	8,502,528,820
Total bases uniquely mapped	7,330,875,865	6,294,992,574	5,842,891,532
Total bases mapping to target	5,449,744,981	4,422,564,459	4,033,208,506
Mean target coverage	109.336672	88.706873	81.004891
% target bases with ≥10x coverage	85.51%	85.04%	83.56%

**Table 2:** Filtered variations

**Chromosome**	**Position**	**Region**	**Gene**	**Function**	**dbSNP132**	**1000G_2010Nov_ALL**	**PolyPhen2**	**SIFT**	**ref_allele**	**alter_allele**	**Proband**	**Father**	**Mother**
chr1	223,175,758	exonic	*DISP1*	nonsynonymous SNV	-	-	1	0	G	A	△	×	×
chr1	240,371,141	exonic	*FMN2*	nonsynonymous SNV	-	-	0.76515	0	C	T	△	×	×
chr1	247,615,260	exonic	*OR2B11*	Deletion and frameshift > premature terminal codon	-	-			A	-	△	×	×
chr2	189,860,860	exonic	*COL3A1*	nonsynonymous SNV	-	-	0.784653	0	G	A	△	×	×
chr2	190,718,672	exonic	*PMS1*	nonsynonymous SNV	-	-	0.996	0	G	A	△	×	×
chr6	30,861,156	exonic	*DDR1*	nonsynonymous SNV	-	-	0.16	0.03	G	A	△	×	×
chr6	105,824,051	exonic	*PREP*	nonsynonymous SNV	-	-	0.241	0	C	T	△	×	×
chr8	113,277,807	exonic	*CSMD3*	nonsynonymous SNV	-	-	0.985	0	C	T	△	×	×
chr11	124,267,132	exonic	*OR8B3*	nonsynonymous SNV	-	-	0.275	0.02	A	G	△	×	×
chr12	49,580,541	exonic	*TUBA1A*	nonsynonymous SNV	-	-	0.777136	0	C	G	△	×	×
chr16	75,682,281	exonic	*TERF2IP*	nonsynonymous SNV	-	-	0.214	0.02	G	A	△	×	×
chr17	74,288,545	exonic	*QRICH2*	nonsynonymous SNV	-	-	0.995	0.01	C	G	△	×	×
chr19	1,055,329	exonic	*ABCA7*	nonsynonymous SNV	-	-	0.996	0	A	G	△	×	×
chr21	10,942,950	exonic	*TPTE*	nonsynonymous SNV	-	-	0.999	0.01	T	C	△	×	×
chr22	25,011,077	exonic	*GGT1*	nonsynonymous SNV	-	-	0.318	0.01	C	T	△	×	×
chrX	153,008,788	exonic	*ABCD1*	nonsynonymous SNV	-	-	1	0	G	A	△	×	×

ref, reference; alter, alteration; exonic, exonic region; SNV, single nucleotide variation; △, heterozygous; ×, no SNV.

**Figure 3:**
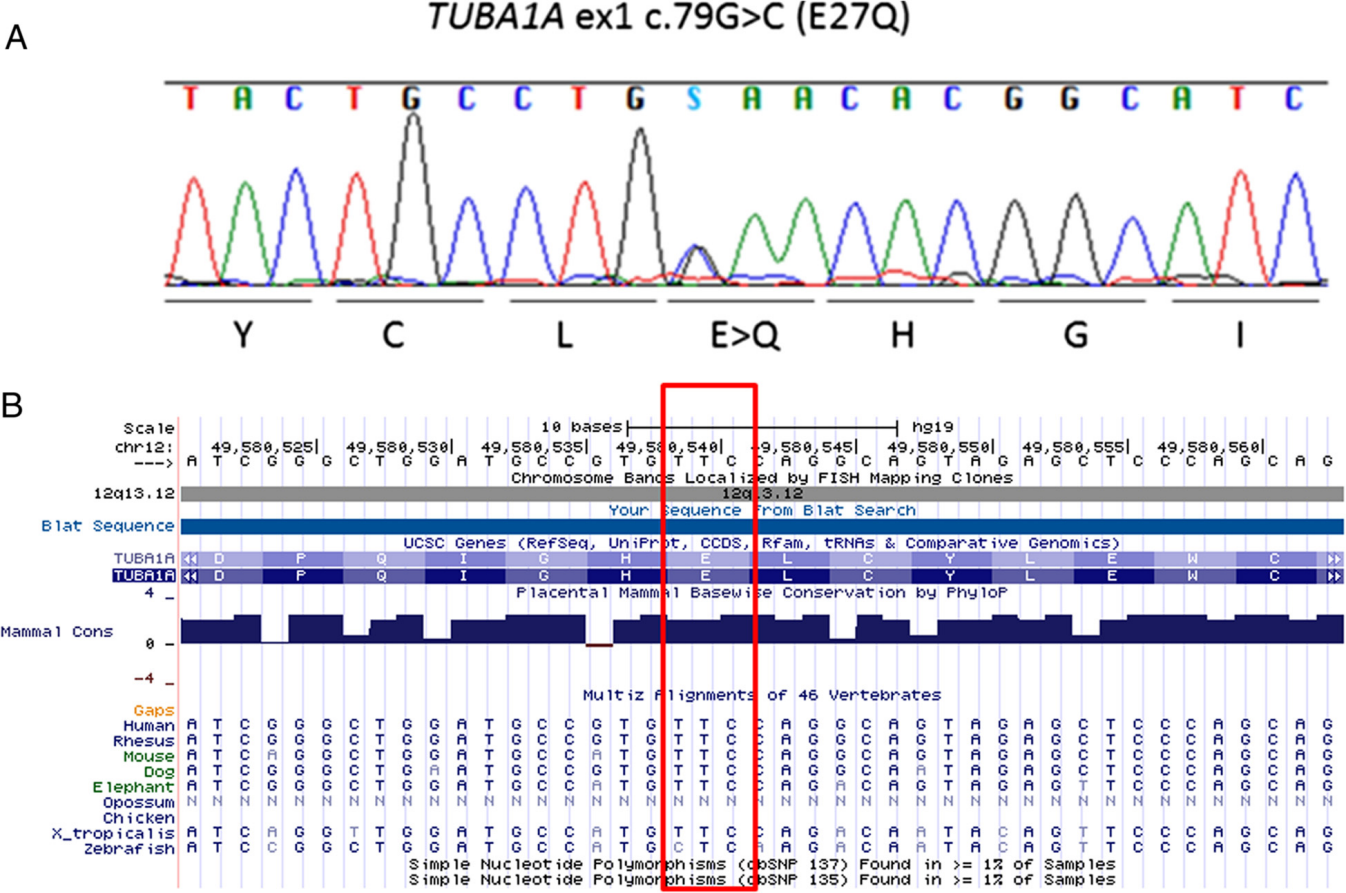
**Results of Sanger sequencing and a map of the identified mutation locus. (A)** A Sanger sequencing electropherogram identifying the missense mutation, c.79G > C (E27Q), is shown. **(B)** A genome map of the locus surrounding the mutation captured from the UCSC genome browser is depicted. Because *TUBA1A* is encoded in an antisense manner, the reverse-complement nucleotide sequence is shown. The affected amino acid is indicated by a red rectangle. The c.79C residue is conserved among many species as shown.

Among the 16 candidate variants in the *de novo* mutation model, a *TUBA1A* variant, E27Q, was included. The damaging effects of the variations were scored by PolyPhen-2 and SIFT; the *TUBA1A* E27Q variant showed a possible damaging effect. We considered the *TUBA1A* variant as the strongest candidate mutation. Subsequent Sanger sequencing confirmed the presence of this variant in the proband and absence in the genomes of both parents (Figure 3A). No variants in the other candidate genes related to MCD were observed. Therefore, we concluded that MCD in this patient was the consequence of a *de novo* mutation in *TUBA1A*. The risk of recurrence in this family was estimated to be the same as that for the general population.

## Discussion

MCD is the consequence of abnormal neuronal migration, which results in intellectual impairment and epilepsy [11]. Many of the genes responsible for MCD are related to autosomal dominant traits caused by *de novo* mutations—e.g., mutations in *PAFAH1B1*, *TUBA1A*, *TUBB2B*, and *TUBB3*. However, some genes are related to autosomal recessive MCD phenotypes, including the genes encoding reelin (*RELN*), very-low-density lipoprotein receptor (*VLDLR*), tubulin alpha-8 (*TUBA8*), as well as X-linked genes encoding doublecortin (*DCX*), aristaless related homeobox (*ARX*), and filamin A alpha (*FLNA*) [1,12,13]. Therefore, molecular diagnostic testing in patients with MCD is an important prerequisite for genetic counseling, because the risk of recurrence cannot be estimated in cases that lack a definitive molecular diagnosis.

In this study, we successfully identified a *de novo TUBA1A* mutation in a patient with sporadic MCD using whole-exome sequencing of trio samples. Among the genes in the tubulin family, *TUBA1A* was the first to be identified as a causative factor of MCD. The TUBA1A protein is thought to function as a scaffold protein [14].

Although there are at least 34 known *TUBA1A* mutations, and many of these mutations are located in the C-terminal half of the amino acid sequence [3,11,14-27], this report is the first to describe the E27Q mutation (Additional file 1: Figure S1). In contrast to most of the previously reported mutations, this mutation is located in the N-terminal region (Additional file 1: Figure S1). Because the mutation is located in a conserved region of tubulin (Additional file 1: Figure S1), and the mutated residue is conserved among many species (Figure 3B), we predicted that this mutation might be pathogenic. We analyzed the effects of this mutation by using web-based tools to predict the secondary structure of the mutated protein. Of the two different computational simulations, one predicted a significant change in the structure of TUBA1A (Additional file 2: Figures S2 and Additional file 3: Figure S3), suggesting that this mutation is likely pathogenic.

Amino acid substitutions may affect heterodimer stability and/or microtubule dynamics by altering the structure of tubulin during nucleotide exchange and hydrolysis. Microtubule stability may be diminished during the critical process of cell migration [28]. All mutations reported thus far in *TUBA1A*, *TUBB2B*, and *TUBB3* are heterozygous missense mutations [3,4,29-31]. Missense mutations in the absence of nonsense mutations, frameshifts, or genomic deletions support altered protein function, rather than haploinsufficiency, as the primary genetic etiology of these tubulin-related disorders [28].

Retrospectively, we evaluated the MRI findings of this patient. Brain MRI showed a colpocephalic appearance of the lateral ventricle dilatation associated with a simplified gyral pattern. Hypoplastic corpus callosum and cerebellar vermis hypoplasia were also noted. According to the classification proposed by Kumar et al. [11], lissencephaly with cerebellar hypoplasia (LCH) in group 3 would be most compatible with this patient, because of the involvement of the corpus callosum and cerebellum. Ross et al. proposed genotype-phenotype correlations in LCH cases encompassing heterogeneous disorders such as those classified as LCH types a—d [32]. LCHa cases are related to mutations in *PAFAH1B1* or *DCX*, whereas LCHb cases are linked to a mutation in *RELN*. Based on the LCH pattern of the patient presenting microcephaly (≤ - 3 SD), this case may be classified as LCHd, which could be related to a mutation in *TUBA1A*. Therefore, no contradiction in the etiology of the *TUBA1A* mutation was observed.

Generally, young couples whose first child has been afflicted with a sporadic case of severe disease are eager to have a healthy child in their next pregnancy [33]. Therefore, a precise genetic diagnosis is necessary. However, many MCD candidate genes have been identified, and Sanger sequencing for predicted causative genes is rather expensive, laborious, and time-consuming [34]. In comparison, in this study, we show that whole-exome sequencing is rapid and comprehensive [35]. We further emphasize that the results of this method provided us not only with confirmation of the causative genes but also simultaneously rules out mutations in other candidate genes. Therefore, we could completely negate the contribution of other candidate genes in this patient. In the future, targeted panel sequencing rather than whole-exome sequencing may become the standard method used to evaluate human disorders derived from multiple candidate genes.

## Conclusion

We successfully identified a causative *TUBA1A* mutation in a patient with sporadic MCD associated with a simplified gyral pattern by using whole-exome sequencing. The identified novel mutation (E27Q) was located in the N-terminal region of the amino acid sequence. Rapid and comprehensive mutation analysis by using whole-exome sequencing may be useful for genetic counseling in sporadic cases of human disorders derived from multiple candidate genes.

## Consent

Written informed consent was obtained from the patient’s parents for publication of this Case Report and any accompanying images. A copy of the written consent is available for review by the Editor-in-Chief of this journal. Blood samples from the patient and her biological parents were obtained with parental consent. This study was approved by the ethical committee at our institution.

## Abbreviations

MCD: Malformations of cortical development; LCH: Lissencephaly with cerebellar hypoplasia; PAFAH1B1: Platelet-activating factor acetylhydrolase isoform 1b regulatory subunit 1; TUBA1A: Tubulin alpha 1a; TUBB2B: Tubulin beta 2B class IIb; TUBB3: Tubulin beta 3 class III; SD: Standard deviation; MRI: Magnetic resonance imaging; SNV: Single-nucleotide variant; indel: Insertions and deletion; PCR: Polymerase chain reaction; RELN: Reelin; VLDLR: Very-low-density lipoprotein receptor; TUBA8: Tubulin alpha-8; DCX: Doublecortin; ARX: Aristaless related homeobox; FLNA: Filamin A alpha; LCH: Lissencephaly with cerebellar hypoplasia.

## Competing interests

The authors declare that they have no competing interest.

## Authors’ contributions

KS analyzed and interpreted the data, evaluated the genotype-phenotype correlation, and drafted the paper. TF performed whole-exome sequencing. AS analyzed the sequencing data. AN and YM correlated clinical information. TY designed the study and reviewed this article. All authors read and approved the final manuscript.

## Supplementary Material

Additional file 1: Figure S1 Location of the identified *TUBA1A* mutation and a comparison of the *TUBA1A* amino acid sequence with tubulin family members. (Top) Although many mutations are located in the C-terminal region, the mutation identified in this patient (E27Q; indicated by red characters), is located in the N-terminal region. (Bottom) E27 is conserved among tubulin family member (red rectangle). Click here for file

Additional file 2: Figure S2 Predicted conformational changes in the protein structure caused by the identified mutation. The Pôle Bioinformatique Lyonnais Network Protein Sequence Analysis predicted a conformational change caused by the E27Q mutation. The location of the mutation (E27Q) is indicated by a red arrow. Click here for file

Additional file 3: Figure S3 The predicted secondary structure of TUBA1A and a simulation of the conformational change. The PSIPRED Protein Sequence Analysis Workbench predicted no definite change between the wild-type and mutant protein. Click here for file
