# Supplementary figures and images for: Whole-exome sequencing identifies a de novo TUBA1A mutation in a patient with sporadic malformations of cortical development: a case report
Source: BMC Res Notes. 2014 Jul 22;7:465. doi: 10.1186/1756-0500-7-465 (PMC4118784; doi:10.1186/1756-0500-7-465)

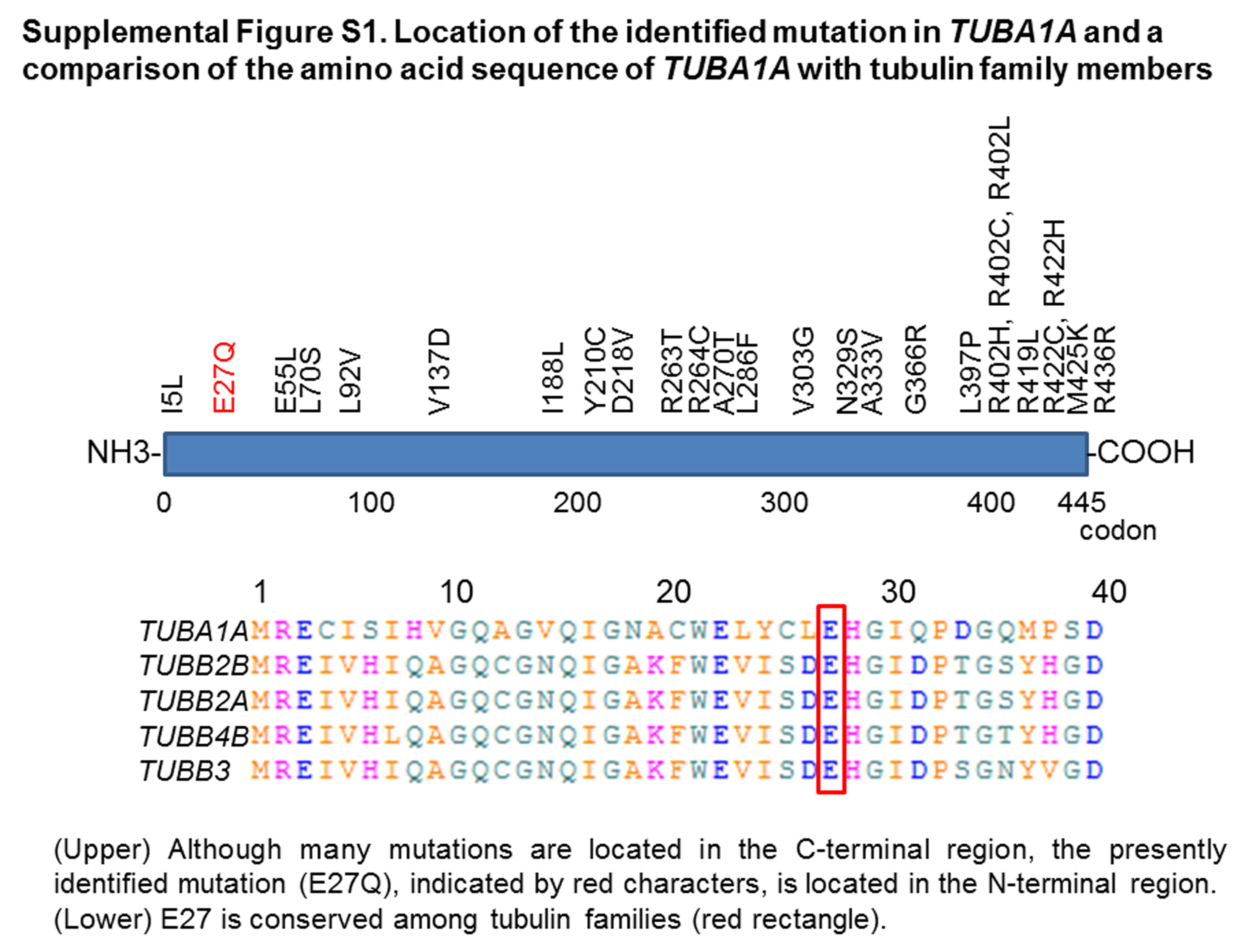

Supplement: Additional file 1: Figure S1 — Location of the identified TUBA1A mutation and a comparison of the TUBA1A amino acid sequence with tubulin family members. (Top) Although many mutations are located in the C-terminal region, the mutation identified in this patient (E27Q; indicated by red characters), is located in the N-terminal region. (Bottom) E27 is conserved among tubulin family member (red rectangle). [file 1756-0500-7-465-S1.tiff]

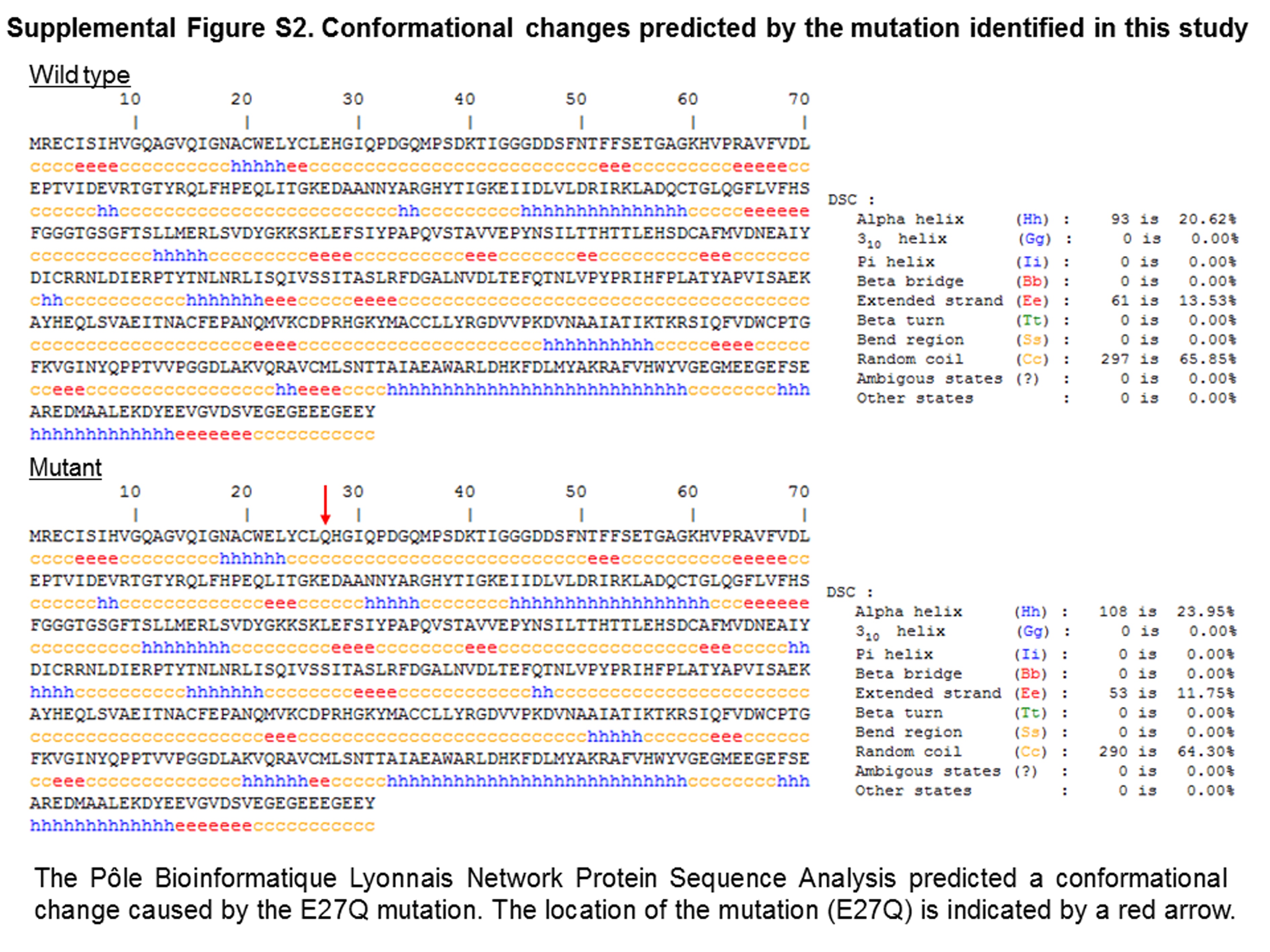

Supplement: Additional file 2: Figure S2 — Predicted conformational changes in the protein structure caused by the identified mutation. The Pôle Bioinformatique Lyonnais Network Protein Sequence Analysis predicted a conformational change caused by the E27Q mutation. The location of the mutation (E27Q) is indicated by a red arrow. [file 1756-0500-7-465-S2.tiff]

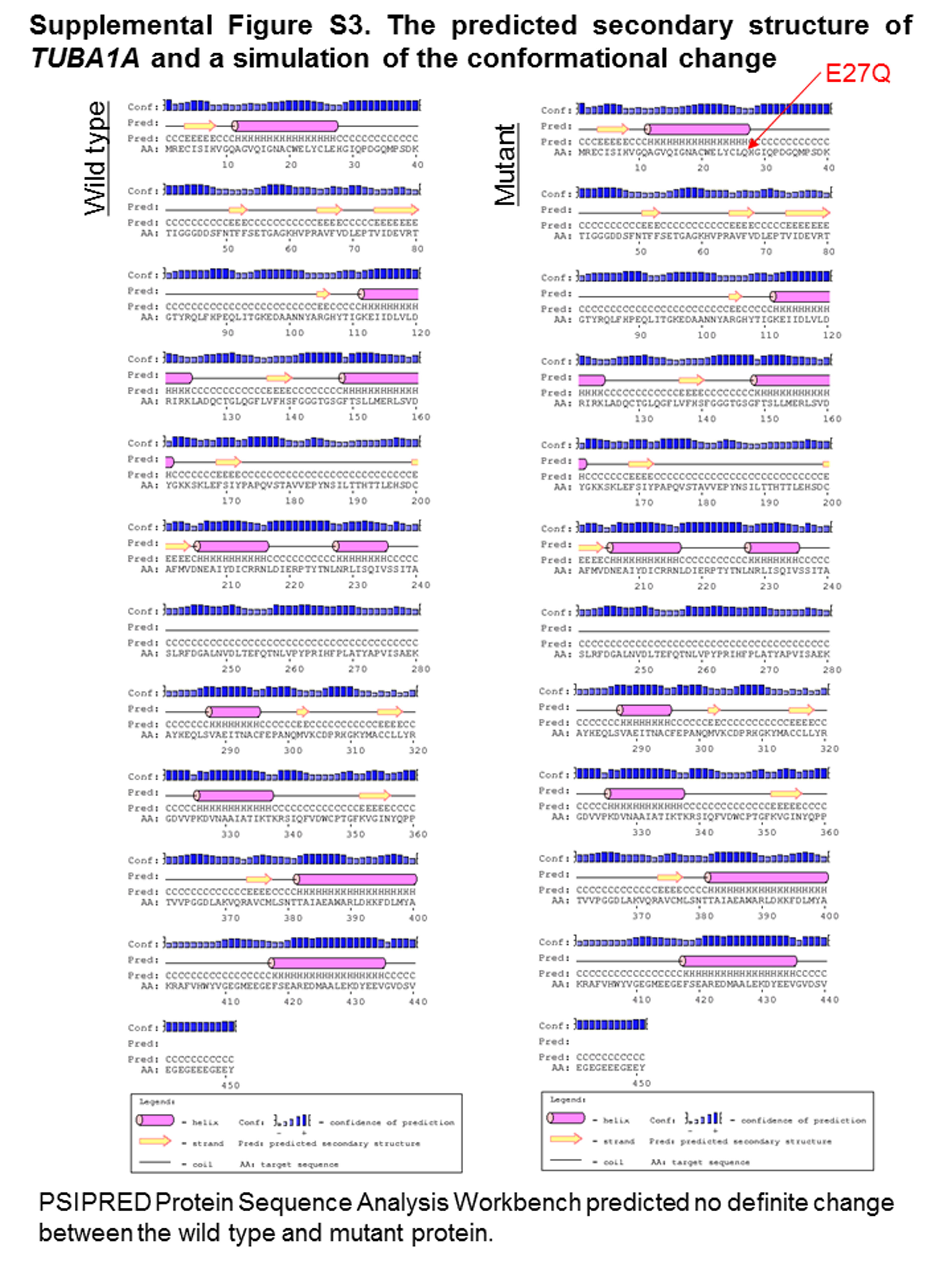

Supplement: Additional file 3: Figure S3 — The predicted secondary structure of TUBA1A and a simulation of the conformational change. The PSIPRED Protein Sequence Analysis Workbench predicted no definite change between the wild-type and mutant protein. [file 1756-0500-7-465-S3.tiff]
